# Urine collection devices to reduce contamination in urine samples for diagnosis of uncomplicated UTI: a single-blind randomised controlled trial in primary care

**DOI:** 10.3399/BJGP.2021.0359

**Published:** 2022-03-25

**Authors:** Gail Hayward, Sam Mort, Ly-Mee Yu, Merryn Voysey, Margaret Glogowska, Caroline Croxson, Yaling Yang, Julie Allen, Johanna Cook, Sarah Tearne, Nicola Blakey, Sharon Tonner, Vanshika Sharma, Meena Patil, Sadie Kelly, Christopher C Butler

**Affiliations:** Nuffield Department of Primary Care Health Sciences, University of Oxford, Oxford.; Nuffield Department of Primary Care Health Sciences, University of Oxford, Oxford.; Nuffield Department of Primary Care Health Sciences, University of Oxford, Oxford.; Nuffield Department of Primary Care Health Sciences, University of Oxford; Oxford Vaccine Group, Department of Paediatrics, University of Oxford, Oxford.; Nuffield Department of Primary Care Health Sciences, University of Oxford, Oxford.; Nuffield Department of Primary Care Health Sciences, University of Oxford, Oxford.; Nuffield Department of Primary Care Health Sciences, University of Oxford, Oxford.; Nuffield Department of Primary Care Health Sciences, University of Oxford, Oxford.; Nuffield Department of Primary Care Health Sciences, University of Oxford, Oxford.; Institute of Applied Health Research, University of Birmingham, Birmingham.; Nuffield Department of Primary Care Health Sciences, University of Oxford, Oxford.; Nuffield Department of Primary Care Health Sciences, University of Oxford, Oxford.; Nuffield Department of Primary Care Health Sciences, University of Oxford, Oxford.; Nuffield Department of Primary Care Health Sciences, University of Oxford, Oxford.; Nuffield Department of Primary Care Health Sciences, University of Oxford, Oxford.; Nuffield Department of Primary Care Health Sciences, University of Oxford, Oxford.

**Keywords:** diagnosis, laboratory analysis, randomised controlled trial, urinary tract infections, urine specimen collection, general practice

## Abstract

**Background:**

Urine collection devices (UCDs) are being marketed and used in clinical settings to reduce urine sample contamination, despite inadequate supporting evidence.

**Aim:**

To determine whether UCDs, compared with standardised instructions for urine sample collection, reduce the proportion of contaminated samples.

**Design and setting:**

Single-blind randomised controlled trial in general practices in England and Wales.

**Method:**

Women aged ≥18 years presenting with symptoms attributable to urinary tract infection (UTI) were randomised (1:1:1) to use either a Peezy UCD or a Whiz Midstream UCD, or were given standardised verbal instructions (SVI) for midstream sample collection. The primary outcome was the proportion of urine samples reported as contaminated by microbiology laboratory analysis.

**Results:**

A total of 1264 women (Peezy UCD: *n* = 424; Whiz Midstream UCD: *n* = 421; SVI: *n* = 419) were randomised between October 2016 and August 2018. Ninety women were excluded from the primary analysis as a result of ineligibility or lack of primary outcome data, leaving 1174 (Peezy UCD: *n* = 381; Whiz Midstream UCD: *n* = 390; SVI: *n* = 403) for intention-to-treat analysis. The proportion of contaminated samples was 26.5% with the Peezy UCD, 28.2% with the Whiz Midstream UCD, and 29.0% with SVI (relative risk: Peezy UCD versus SVI = 0.91, 95% CI = 0.76 to 1.09, *P* = 0.32; Whiz Midstream UCD versus SVI = 0.98, 95% CI = 0.97 to 1.20, *P* = 0.82). There were 100 (25.3%) device failures with the Peezy UCD and 35 (8.8%) with the Whiz Midstream UCD; the proportion of contaminated samples was similar after device failure samples were excluded.

**Conclusion:**

Neither the Peezy UCD nor the Whiz Midstream UCD reduced urine sample contamination when used by women presenting to primary care with suspected UTI. Their use cannot be recommended for this purpose in this setting.

## INTRODUCTION

Urinary tract infection (UTI) is one of the most common bacterial infections managed in general practice and is the reason for 1%–3% of all GP consultations.^1^ UTI is more common in women, for whom the lifetime risk is 50% and annual incidence is >10%.^1^ Urine culture, a test which can take up to 3 days to provide a result, is commonly requested by primary care clinicians to confirm a suspected diagnosis of UTI and to understand antibiotic sensitivities. In the UK, some laboratories perform urine culture on all urine samples, whereas others can use the concentration of white cells, determined using flow cytometry, to identify samples that will proceed to urine culture.

Uniquely among medical tests, GPs will not receive a clinically useful result from the urine culture in up to 30% of cases because these samples are contaminated by bacteria from the host’s faeces, skin, and/or vaginal secretions.^2^ Such samples are often reported as contaminated or as ‘mixed growth’, and this result neither rules in nor rules out bacterial infection. Repeat samples are often required in order to guide care, with implications for patients, who may have to wait longer for appropriately targeted antibiotic therapy, and for health services because repeated culture tests are costly and time consuming.

Few solutions have been proposed for this wide-scale problem. Patients have been advised to discard the first part of the urine stream, as this may contain most of the contaminants, and capture only the mid-stream for the sample.^3^^,^^4^ However, this procedure is often challenging to explain and to implement, with two small low-quality randomised trials finding no clear evidence of benefit.^5^

Another solution, which is already being implemented in healthcare settings, is the use of a urine collection device (UCD). The Whiz Midstream (Oxford Devices, Oxford)^6^ uses a pressure valve system to allow the early urine stream to flow into the toilet and the midstream sample then flows into a sample bottle. The Peezy (Forte Medical, London)^7^ device channels the early stream into a sponge, collecting the sample by back pressure. However, of three published studies evaluating the impact of UCD use on urine culture contamination,^8^^–^^10^ only one, using the Whiz Midstream UCD in antenatal women with no UTI symptoms,^8^ found evidence of small benefit. No randomised trial has been conducted in the population with greatest potential for widespread benefit, that is, women presenting to primary care with uncomplicated UTI. This study therefore aimed to investigate the effectiveness of UCDs in reducing the proportion of urine samples from women with suspected uncomplicated UTI that are contaminated on laboratory culture.

**Table:** How this fits in

To the authors’ knowledge, this is the first trial to evaluate the effectiveness of urine collection devices (UCDs) in the population of most relevance: women with symptoms of urinary tract infection (UTI) presenting to primary care. Neither the Peezy UCD nor the Whiz Midstream UCD reduced sample contamination when used by women presenting to primary care with symptoms attributable to uncomplicated UTI. Therefore, their use cannot be recommended for this purpose in this setting.

## METHOD

A three-arm, single-blind, individually randomised controlled trial was conducted. The trial was registered with the ISRCTN registry (reference: ISRCTN68511881). Participating practices in England and Wales responded to an advertisement for expressions of interest from their local Clinical Research Network. GPs and nurses were responsible for patient recruitment.

### Participants

Women aged ≥18 years presenting to UK general practice between October 2016 and August 2018 were eligible for inclusion if they had symptoms attributable to uncomplicated UTI, including at least one of dysuria, haematuria, or frequency of urination, and were able to give informed consent. Exclusion criteria were current or recent (<7 days) antibiotic use, indwelling catheter or intermittent self-catheterisation, previous recruitment to this trial or current involvement in a clinical trial of an investigational medicinal product, inability to provide a sample during the index consultation, or to understand and complete trial materials in English.

### Interventions

Participants were randomised to obtain a urine sample using one of the following approaches:

- Standardised verbal instructions (SVI) for collecting a midstream urine without a UCD as follows: ‘Please pass the first portion of your urine into the toilet and collect the next portion in this sample pot.’
- Peezy UCD with instructions for its use on the device packaging.
- Whiz Midstream UCD with instructions for its use on the device packaging.

Devices were purchased by the trial team using usual purchasing routes. UCD manufacturers did not provide any funding towards the study and had no role in its design, conduct, or analysis.

### Study procedures

After eligibility assessment and written informed consent, baseline questionnaires and case report forms (CRFs) were completed. Participants who were unable to pass urine during their baseline appointment were deemed ineligible post-randomisation. If the participant was able to pass urine and attempted to collect a sample using a device but was unable to do so (a device failure), they continued in the study and were offered a standard collection pot to attempt to produce a second sample. Because participants were generally unwilling to attempt to use a device for a second time this pragmatic approach was decided on after discussion with the study Patient and Public Involvement group. Since device failures had not been anticipated to be frequent based on previous studies,^8^^,^^9^ recruited general practices were initially trained to report device failures through direct telephone or email contact with the study team. From 19 May 2017 the baseline CRF allowed documentation of device failure as this was more frequent than anticipated.

Participants were contacted by telephone or text message at 14 days to collect information on their symptom duration and healthcare usage, and to complete a EQ-5D-5L health questionnaire. At primary care record review at 14 days, antibiotic prescriptions and healthcare contacts were documented.

To assess the proportion of participants who were approached who then enrolled in the study, recruiting practices were asked to provide detailed data over a 2-week ‘snapshot’ period on the number of participants who were approached, who were eligible for inclusion in the study, and who were recruited. This was done because, in the authors’ experience, continuous screening logs have historically been very poorly completed.

### Randomisation and blinding

Participants were randomised (1:1:1) using Sortition, an online clinical trial randomisation system developed and managed by the Clinical Trials Unit in the Nuffield Department of Primary Care Health Sciences, University of Oxford. The laboratory staff reporting the primary outcome and the trial team entering participant data were blinded to intervention allocation, but the patient and recruiting clinician were not. Patient follow-up data were collected by trial administrators who were trained to avoid being informed of the participant’s allocation as far as possible. Trial statisticians were blind to group allocation.

### Outcome measures

The primary outcome was the proportion of contaminated urine samples, defined as those reported as a mixed growth according to NHS laboratory national standard operating procedures. Secondary outcomes were the proportion of samples reported as a pure or predominant growth of a known urinary pathogen according to national standard reporting procedures (the actionable information of most importance to clinicians), the presence and number of white cells (often elevated in UTI) and epithelial cells (skin cells, which can also indicate sample contamination) on urine microscopy, the diagnostic accuracy of dipstick urinalysis for a pure or predominant growth of a known urinary pathogen on urine culture, healthcare resource use, duration of symptoms, and health utility measured with the EQ-5D-5L health questionnaire (to be reported separately, along with tertiary microbiological outcomes). Hospitalisations related to UTI within 14 days of baseline assessment were recorded.

### Sample handling

The recruiting clinician in each practice performed a dipstick urinalysis and sent the sample to a central study laboratory. If the responsible GP required a culture result for clinical care, the sample was split into two. The study sample was cultured and reported using standard NHS laboratory procedures and definitions. Where sufficient volumes of urine were available, flow cytometry (measured with sediMAX automated urine microscopy analyser) was used to identify and quantify epithelial cells and white cells.

### Sample size calculation

Allowing for a 5% loss of samples, it was estimated that a sample size of 1191 participants would provide 90% power to detect an absolute reduction of contaminated samples of 10.8%. Although the proportion of urine samples reported as contaminated from primary care nationally is not known, the proportion in the authors’ local tertiary care hospital microbiology laboratory averaged 26% between 2008 and 2018.^11^ A reduction of <10% in the proportion of contaminated samples was not felt to be clinically meaningful.

### Statistical analysis

The primary analysis population was defined as all participants for whom data were available and were analysed according to the groups they were randomly allocated to, regardless of device failures and deviation from protocol. For the primary outcome, and other binary outcomes (reporting of samples, presence of white blood and epithelial cells, requiring ≥1 further urine cultures, and requiring a repeat consultation with GP or hospital admission), a generalised linear mixed-effects model analysed all data available, adjusting for intervention arm as a fixed effect, and GP surgery as a random effect. An ordinal logistic mixed-effects regression model was used to analyse the concentration of white blood and epithelial cells. Duration of symptoms was analysed by survival analysis in a mixed-effects Cox proportional hazards model, censoring on whether symptoms resolved or not. Sensitivities and specificities of the dipstick urinalysis (assuming a pure growth of a known urinary pathogen from urine culture as reference standard) were evaluated using a decision rule as follows: a positive dipstick result (indicating presence of UTI) was indicated by the dipstick result showing nitrite (positive), or both leucocytes (+, ++, +++) and blood (haemolysis trace or greater).^12^ A second analysis used only the presence of leucocytes to indicate infection. Sensitivities and specificities were compared by means of a Fisher’s exact test.

The primary outcome was obtained from the reporting of the urine culture from the central laboratory. Where the primary outcome was not available from the central laboratory, but the GP had split the sample in order to receive a result to guide clinical care, this NHS laboratory result was available from medical notes review and used as the primary outcome. A sensitivity analysis analysed only the culture results from the research sample provided for the study. Since NHS laboratories can use different procedures, a post-hoc sensitivity analysis was performed using only the trial samples or split NHS samples that were analysed in the central laboratory.

A per-protocol sensitivity analysis was performed on the primary outcome on only those women who provided a sample with the allocated device. All women who reported experiencing a device failure were excluded from the per-protocol analysis.

Planned subgroup analyses were performed for women who were post-menopausal, pregnant, or who had a history of recurrent UTI. These groups are suggested to have physiological changes in the urinary tract,^13^^–^^15^ which might have the potential to alter either the proportion of samples that are contaminated or device performance. Samples received ≤48 hours and >48 hours after provision were compared to explore the potential impact of duration of storage and transport on the findings.

### Patient and public involvement

Four women who had experienced UTI supported this project from inception to dissemination, providing feedback on study design and materials. They advised on processes for participants and recruitment, and dissemination of findings. One member of this group served on the trial steering committee.

## RESULTS

**Figure 1.**
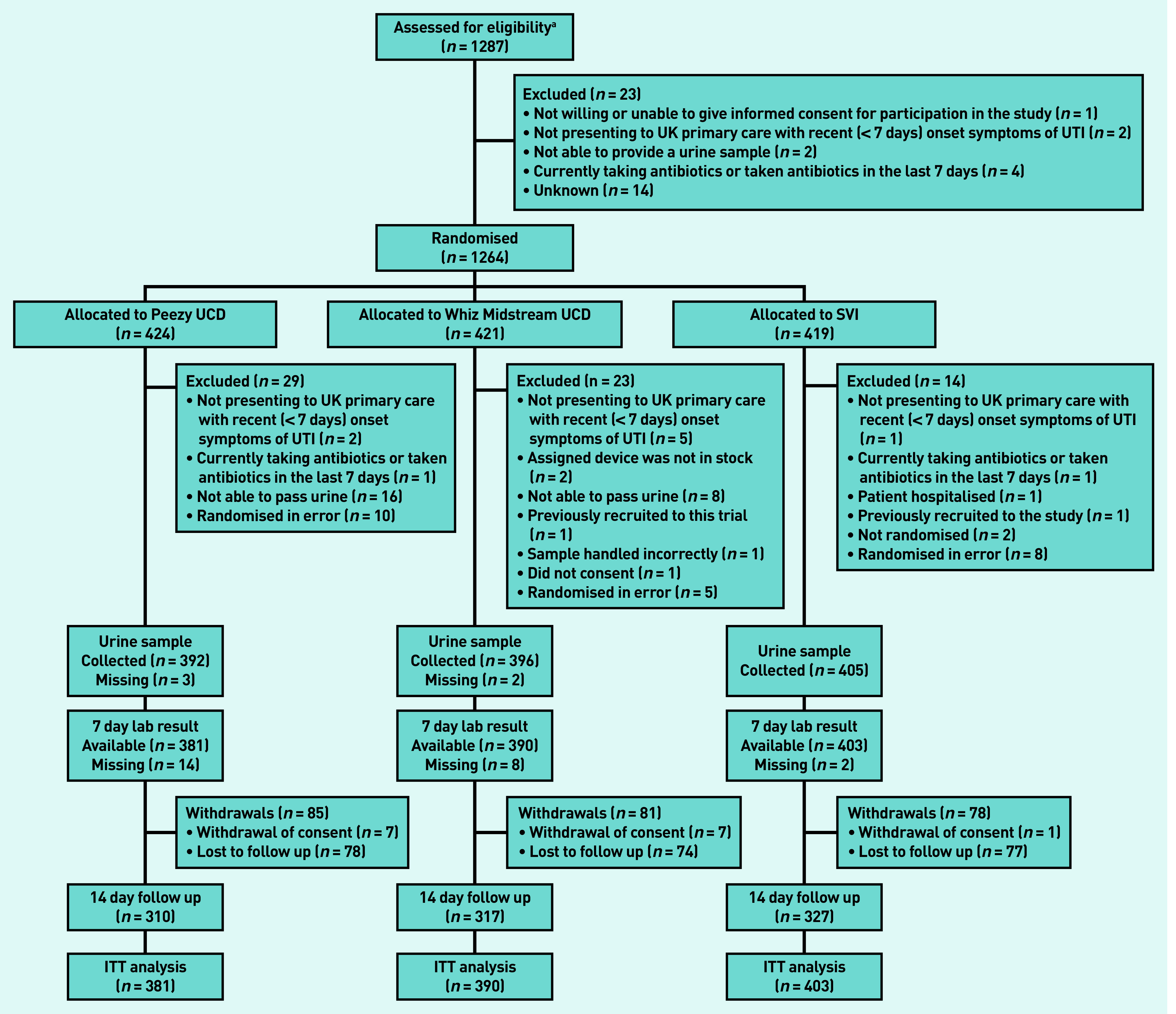
***Consort flow diagram.*** *^a^****A 2-week snapshot screening log suggested that 63.9% of patients approached were eligible and 39.6% of those approached consented to participate (63.6% of those eligible). ITT = intention to treat. SVI = standardised verbal instructions. UCD = urine collection device. UTI = urinary tract infection.***

From October 2016 to August 2018, 1264 women from 61 general practices in England and Wales were randomised. A total of 424 participants were randomised to the Peezy UCD, 421 to the Whiz Midstream UCD, and 419 to SVI. Figure 1 gives details of participant screening, recruitment, and follow-up, and reasons for ineligibility after randomisation. Of the participants, 29 (Peezy UCD), 23 (Whiz Midstream UCD), and 14 (SVI) were found to be ineligible subsequent to randomisation. Sixteen participants in the Peezy UCD arm and eight in the Whiz Midstream UCD arm were ineligible as they were unable to pass urine; no patients in the SVI arm were excluded for this reason. This meant that 1174 (Peezy UCD: *n* = 381; Whizaway UCD: *n* = 390; SVI: *n* = 403) participants remained for the intention-to-treat analysis. Device failures, for example, caused by urine failing to enter the specimen tube, urine failing to enter the device as a whole, or parts of the device falling into the toilet, were reported by 25.3% (*n* = 100/395) of participants using a Peezy UCD and by 8.8% (*n* = 35/398) of participants using a Whiz Midstream UCD. Five participants (three using the Peezy UCD and two the Whiz Midstream UCD) experienced a device failure and were unable to provide a second urine specimen in a standard container. There were 775 samples (253 Peezy UCD, 206 Whiz Midstream UCD, and 316 SVI) of sufficient volume to allow flow cytometry analysis (Table 2).

**Table 2.** Proportion of samples containing and RR of a pure or predominant growth of a uropathogen, proportion containing and RR of white cells or epithelial cells, and the diagnostic accuracy (sensitivity and specificity) of urine dipstick analysis

**Result**	**Peezy UCD**	**Whiz Midstream UCD**	**Standardised verbal instructions**
**Urine culture results, *N***	**395**	**398**	**405**
Pure or predominant growth, *n* (%)	194 (55.0)	197 (52.8)	216 (54.4)
No predominant growth, *n* (%)	159 (45.0)	176 (47.2)	181 (45.6)
Missing, *n*	42	25	8
Adjusted RR of pure or predominant growth	1.01	0.97	Ref
95% CI	0.86 to 1.18	0.88 to 1.07	—
*P*-value	0.900	0.558	—

**Presence of cells in urine samples detected using flow cytometry, *N***	**253**	**206**	**316**
Epithelial cells, *n* (%)	114 (45.1)	117 (56.8)	174 (55.1)
Adjusted RR of presence of epithelial cells	0.61	0.87	Ref
95% CI	0.45 to 0.83	0.71 to 1.33	—
*P*-value	0.001	0.865	—
White cells, *n* (%)	142 (56.1)	114 (55.3)	203 (64.2)
Adjusted RR of presence of white cells	0.87	0.86	Ref
95% CI	0.76 to 1.00	0.74 to 1.00	—
*P*-value	0.052	0.047	—
Missing, *n*	142	192	89

**Diagnostic accuracy of dipstick urinalysis^a,b^**			
Sensitivity, % (95% CI)	59.79 (52.53 to 66.75)	63.45 (56.31 to 70.18)	65.28 (58.52 to 71.61)
Specificity, % (95% CI)	62.89 (54.88 to 70.41)	63.07 (55.48 to 70.21)	60.77 (53.26 to 67.93)

*Log-Poisson generalised linear mixed effect model of presence of pure or predominant growth, white blood cells, or epithelial cells modelled against intervention arm as fixed effects, and GP practice as a random effect. Level of significance = 0.025.*

a *Pure or predominant growth (104–105 or* >*105) of uropathogen on urine culture as reference standard. Decision rule for urine dipstick: a positive dipstick result (indicating presence of urinary tract infection) was indicated by the dipstick result showing nitrite (positive), or both leucocytes (+, ++, +++) and blood (haemolysis trace or greater).^12^*

b *All Fisher’s exact tests statistics* P>*0.2. RR = relative risk. UCD = urine collection device.*

Most baseline characteristics were similar in the three study groups, including symptom severity, age, and dipstick results. Fewer samples were cloudy in the Peezy UCD arm (38.6%) compared with the SVI arm (52.7%) and Whiz Midstream UCD arm (43.2%), and more participants had a sample additionally sent for routine care in the SVI arm (SVI 52.1%, Peezy UCD 39.3%, and Whiz Midstream UCD 44.0%; see Supplementary Table S1 for details).

### Primary outcome

The proportion of samples reported as contaminated by standard NHS laboratory procedures was not significantly different when comparing either UCD with SVI (Table 1). The relative risk (RR) of contamination with the Peezy UCD compared with SVI was 0.91 (95% confidence intervals [CI] = 0.76 to 1.09, *P* = 0.32) and of the Whiz Midstream UCD was 0.98 (95% CI = 0.97 to 1.20, *P* = 0.82). Findings were similar in a per-protocol analysis including only those participants who provided a sample using the allocated approach (Peezy UCD: RR = 0.91; 95% CI = 0.73 to 1.14, *P* = 0.41; Whiz Midstream UCD: RR = 1.01; 95% CI = 0.82 to 1.24, *P* = 0.96). Findings were also similar in pre-specified sensitivity analyses that assumed all missing outcomes were mixed growth, using multiple imputation to replace missing primary outcomes, and including only participant samples that were analysed at the main study laboratory (see Supplementary Table S2 for details). There was no difference in the proportion of contaminated samples when subgroups of participants who were pre-menopausal/post-menopausal, pregnant, or who had a history of recurrent UTI were examined separately for each UCD versus SVI. Subdividing samples into those received ≤48 hours after provision and >48 hours after provision gave similar results (see Supplementary Figures S1 and S2 for details).

**Table 1.** Primary outcome: number and proportion of contaminated samples, device failures, and RRs of contamination for intention-to-treat and per protocol analysis

**Urine result**	**Peezy UCD**	**Whiz Midstream UCD**	**Standardised verbal instructions**
**Intention-to-treat analysis, *N* ^a^**	**395**	**398**	**405**
Mixed growth, *n* (%)^b^	101 (26.5)	110 (28.2)	117 (29.0)
Not ‘mixed growth’, *n* (%)	280 (73.5)	280 (71.8)	286 (71.0)
Device failures, *n*	100	35	0
Missing, *n*	14	8	2
Adjusted RR of contamination	0.91	0.98	Ref
95% CI	0.76 to 1.09	0.97 to 1.20	—
*P*-value	0.315	0.820	—

**Per-protocol, *N* ^c^**	**295**	**363**	**405**
Mixed growth, *n* (%)^b^	77 (26.3)	104 (29.1)	117 (29.0)
Not ‘mixed growth’, *n* (%)	216 (73.7)	254 (70.9)	286 (71.0)
Missing, *n*	2	5	2
Adjusted RR of contamination	0.91	1.01	Ref
95% CI	0.73 to 1.14	0.82 to 1.24	—
*P*-value	0.405	0.955	—

*Log-Poisson generalised linear mixed effect model of lab culture urine result modelled against intervention arm as fixed effects, and GP practice as a random effect. Level of significance = 0.025.*

a *All participants with a urine culture result available regardless of whether the allocated collection method was used.*

b *Mixed growth = contamination.*

c *All participants with a urine culture result available from the allocated collection method instructions. RR = relative risk. UCD = urine collection device*

### Secondary outcomes

The proportion of samples reported as a pure or predominant growth of uropathogen did not differ significantly between either UCD or SVI groups (Peezy UCD: 55.0% versus SVI 54.4%, RR = 1.01, 95% CI = 0.86 to 1.18, *P* = 0.90; Whiz Midstream UCD: 52.8% versus SVI 54.4%, RR = 0.97, 95% CI = 0.88 to 1.07, *P* = 0.56; Table 2).

Use of either UCD did not affect the proportion of urine samples containing white cells (Table 2), although a reduced concentration of white cells was evident with both UCDs compared with SVI (see Supplementary Table S3 for details). Significantly fewer samples with epithelial cells were produced in the Peezy UCD arm (RR = 0.82, 95% CI = 0.70 to 0.95, *P* = 0.010; Table 2) and the concentration of epithelial cells was lower (adjusted odds ratio 0.61, 95% CI = 0.45 to 0.83, *P* = 0.001) compared with the SVI group (see Supplementary Table S3 for details).

There was no significant difference between either UCD or the SVI arm in the diagnostic accuracy of dipstick urinalysis using a decision rule based on the presence of blood, leucocytes, and nitrites with a positive culture result as a reference standard (Table 2).^12^ A decision rule using only presence of leucocytes demonstrated greater sensitivity but not specificity for UTI in the SVI arm than the Peezy UCD arm (sensitivity: Peezy UCD = 68.04%, 95% CI = 60.98% to 74.54%; SVI = 78.70%, 95% CI = 72.64% to 83.97%, *P* = 0.018) (see Supplementary Table S4 for details).

For those participants where the GP chose to receive a culture result, and therefore this result could have influenced onward care, there was no difference between either UCD or SVI in repeat urine cultures, or healthcare contacts for symptoms or complications of UTI. The duration of symptoms was also similar between arms (Table 3). When data from study participants whose GP did not request a culture result were included in the analysis, findings were similar (see Supplementary Table S5 for details). Five serious adverse events were noted (two for SVI, two for the Whiz Midstream UCD, and one for the Peezy UCD): all were hospital admissions with suspected pyelonephritis, which fully resolved after treatment and were deemed unrelated to the intervention (see Supplementary Table S6).

**Table 3.** Health outcomes for participants where the GP received the culture results by group allocation

**Variable**	**Peezy UCD (*n*= 154)**	**Whiz Midstream UCD (*n*= 172)**	**Standardised verbal instructions (*n*= 210)**
**Requiring a repeat consultation from any source for symptoms or complications of UTI within 14 days**			
Yes, *n* (%)	50 (32.5)	54 (31.4)	70 (33.3)
No, *n* (%)	104 (67.5)	118 (68.6)	140 (66.7)
Missing, *n*	0	0	0

**Duration of symptoms, days**			
Mean, *n* (SD)	5.10 (3.88)	5.57 (3.72)	5.19 (3.09)
Median, *n* (IQR)	4.00 (3.00–7.00)	4.50 (3.00–7.00)	4.00 (3.00–7.00)
Range, *n*	0–28	1–21	1–15
Missing, *n*	52	58	64

**Repeat urine culture requested within 14 days**			
Yes, *n* (%)	18 (12.8)	14 (8.5)	19 (9.3)
No, *n* (%)	123 (87.2)	151 (91.5)	185 (90.7)
Missing, *n*	13	7	6

## DISCUSSION

### Summary

UCDs did not reduce the proportion of urine samples from women presenting to primary care with symptoms of uncomplicated UTI that were reported as contaminated on laboratory culture. Furthermore, a quarter of women allocated to the Peezy UCD arm and almost one in ten allocated to the Whiz Midstream UCD arm were unable to collect a urine sample successfully using the UCD. UCD use did not improve the diagnostic accuracy of dipstick urinalysis for infection and did not alter the proportion of urine samples with white cells present or reported as positive for infection on urine culture. Although samples produced using the Peezy UCD had lower levels of epithelial cells, which have also been seen as a marker of contamination, this has little clinical significance, and a recent retrospective study has questioned the link with contamination.^16^

### Strengths and limitations

To the authors’ knowledge, this is the only adequately powered trial of UCDs, and the only one conducted in the symptomatic primary care population where the problem of urine contamination is most prevalent. This pragmatic study used standard NHS transport and laboratory analysis processes.

However, there are several limitations. First, there were more device failures than were expected, when participants could not successfully capture their urine using the device. About a quarter of Peezy UCDs failed, and this may have impacted the intention-to-treat analysis. However, with nearly 300 women in the Peezy UCD arm per-protocol analysis, this trial, to the authors’ knowledge, remains the largest to examine this question.

Second, as would be the case in everyday care, it was not possible to control the time taken for sample transport and laboratory analysis. This means that the proportion of contaminated samples could have been higher because of bacterial growth during any delay. However, no difference was found in contamination in samples analysed ≤48 hours and >48 hours after provision, and a 2019 trial in an emergency department found no benefit of the Peezy UCD, despite having much shorter times between obtaining the sample and analysis.^10^

Finally, it is possible that many patients do not receive explicit instructions for midstream urine collection. It is not known what the impact on the findings would have been had the control arm participants not been issued with instructions.

### Comparison with existing literature

Previous studies evaluating the impact of UCDs on urine culture contamination found either small or no beneficial effects. In the only randomised controlled trial of the Whiz Midstream UCD,^8^ in 2182 asymptomatic pregnant women, a 5% reduction in mixed growth was demonstrated compared with the control group receiving usual care. The only published randomised controlled trial of the Peezy UCD,^10^ recruiting 1374 adults (27% male) in an emergency department, found no significant difference in contamination compared with using a simple collection pot, and higher failure rates in those using Peezy UCD (5% unable to use the device and 20% producing insufficient urine). A non-randomised study of the Peezy UCD in patients who have had renal transplants using historical controls also demonstrated no difference in the proportion of contaminated samples.^9^

### Implications for research and practice

In comparison with standardised instructions for midstream urine collection, this study found no significant benefit of using either the Peezy or Whiz Midstream UCDs on the proportion of contaminated samples in symptomatic women presenting to primary care. The use of UCDs cannot therefore be recommended for this purpose. Although UCDs did not improve care in this study, further evaluation in patient groups where physical limitations may make standard instructions for midstream urine collection harder to accomplish, may be warranted.
